# Efficacy and safety of transepithelial corneal collagen crosslinking surgery versus standard corneal collagen crosslinking surgery for keratoconus: a meta-analysis of randomized controlled trials

**DOI:** 10.1186/s12886-017-0657-2

**Published:** 2017-12-28

**Authors:** Wenwei Li, Bin Wang

**Affiliations:** Department of Ophthalmology, Tongde Hospital of Zhejiang Province, 234 Gucui Road, Hangzhou, 310012 China

**Keywords:** Keratoconus, Transepithelial corneal collagen crosslinking, Standard corneal collagen crosslinking, Maximum keratometry, Visual acuity, Corneal thickness, Meta analysis

## Abstract

**Background:**

The aim of this study was to evaluate the efficacy and safety of transepithelial corneal collagen crosslinking (transepithelial CXL) versus standard corneal collagen crosslinking (epithelium-off CXL) on keratoconus.

**Methods:**

Eligible studies were identified by systematically searching PubMed, the Cochrane Library and Embase. Topographic parameters, corrected distant visual acuity (CDVA), uncorrected distant visual acuity (UDVA), and corneal thickness (CT) were assessed by the pooled weighted mean differences (WMDs) of the change from baseline to the end of follow up. Quality was assessed according to Cochrane handbook. And we used Review Manager to analysis the included trials.

**Results:**

Three trials involving 244 eyes were evaluated, with 111 eyes in the standard CXL group and 133 eyes in the transepithelial CXL group. The pooled results showed that there were significant differences between the two groups in maximum keratometry (mean difference = 1.05D, 95% CI 0.19 to 1.92, *P* = 0.02)),and the standard CXL is more effective in decreasing the maximum keratometry at least 12 months after operation; the transepithelial CXL group gained more improvement in CDVA (mean difference = −0.07, 95% CI -0.12 to −0.02, *P* = 0.007);there were no significant differences in uncorrected distant visual acuity (UDVA) between the two groups (mean difference = −0.03, 95% CI -0.20 to 0.15, *P* = 0.75). A similar change was found in corneal thickness (mean difference = 4.35, 95% CI -0.43 to 9.13, *P* = 0.07)).

**Conclusions:**

The standard CXL is more effective in decreasing the maximum keratometry than the transepithelial CXL; the transepithelial CXL provided favorable visual outcomes; they both exhibit similar safety.

## Background

Characterized by bilateral, noninflammatory (although being questioned recently) and progressive corneal ectasia, keratoconus affects almost one person in almost 2000 [1]. With progressive corneal thinning, corneal protrusion, progressive irregular astigmatism, corneal fibrosis and visual deterioration, it causes huge economical and healthy problems to the suffers. Although the exact etiology is not well understood, it is commonly believed that the genetic predisposition as well as environmental factors servers the final pathway [2]. For mild cases, astigmatic spectacles and soft contact lenses may be effective; with more advanced cases, rigid contact lenses are needed to improve vision; Corneal collagen cross-linking (CXL) is a promising treatment that may slow or stop the progression of keratoconus. Moreover, CXL may decrease the steepness of the cone and improve uncorrected (UDVA) and corrected (CDVA) distance visual acuities as well as subjective visual symptoms in some cases [3] .

For a long time it has been known that crosslinking decreases the flexibility as well as increases the rigidity of many material [4]. In 2003, Wollensak, firstly, reported twenty-three eyes of 22 patients with moderate or advanced progressive keratoconus underwent the operation of corneal collagen crosslinking. The progression stopped in all eyes and 16 eyes (70%) demonstrated a reduction of the maximal keratometry readings by 2.01 diopters and an improvement of visual acuity in 15 eyes (65%) [5]. Since then many other researchers did similar clinical studies corroborating the similar therapy effects with few complications [6, 7]. The procedure needs to remove the central 7–9 mm of the epithelium and then application of a 0.1% riboflavin 5-phosphate and 20% dextran solution are administrated to the deepithelized surface every 5 min for 30 min followed by exposure to UVA (370 nm, 3 mW/cm 2) radiation for a duration of 30 min with continued application of the above solution every 5 min. After the operation, topical antibiotics and a soft bandage contact lens with good oxygen permeability is given to the patients [5]. However, the deepithelization may bring postoperative pain and serve as a potential source of postoperative infections. Transepithelial CXL avoids the need for epithelial removal, thus circumventing these downsides of epithelium removal. Since the introduction of transepithelial CXL, there has been an increasing number of studies published aiming to assess the therapeutic effects of it [8–13]. Recent studies have suggested that transepithelial CXL should be helpful for keratoconus, but only several articles compare the treatment effects and complications between standard (epithelium-off) CXL and transepithelial CXL [14–19].

Therefore, we conducted a systematic review and meta-analysis to summarize the data from included studies and decide which method might be a better choice for patients. Our primary outcome to determine efficacy was the mean change of maximum keratometry, visual acuity and corneal thickness on thinnest point at least 12 months after operation. To the best of our knowledge, this is the first meta-analysis concentrating to evaluate the efficacy and safety of standard CXL versus transepithelial CXL for keratoconus and it might be useful for surgeons to choose the best option for their patients.

## Methods

Search strategy. Two independent reviewers searched the PUBMED (1950 to July 11, 2017), EMBASE (before 1966 to July 11, 2017), and the Cochrane Library (including the Cochrane Central Register of Controlled Trials, 1800 to July 11, 2017). Our search was performed on July 11, 2017. The databases were searched systematically using the following terms:“Cross-Linking Reagents”, “Reagents, Cross-Linking”, “Crosslinking Reagents”, “Reagents, Crosslinking”, “Bifunctional Reagents”, “Reagents, Bifunctional”, “Cross Linking Reagents”, “Linking Reagents, Cross”, “Reagents, Cross Linking” and “Keratoconus”. The search strategy used both keywords and Medical Subject Headings (MeSH) terms. There were no limits placed on the year or language of publication. We reviewed the titles and abstracts of the search results and retrieved full-text articles if the title or abstract appeared to meet the eligibility criteria for this review.

Study Criteria and Outcomes. All publications were screened by two authors according to the following selection criteria independently. Any disagreement was discussed by the two authors and resolved. The inclusion criteria used in the present meta-analysis were as follows: (1) study design: randomized or nonrandomized clinical trials; (2) population: patients with keratoconus; (3) intervention: transepithelial CXL versus epithelium-off CXL; and (4) outcome variables: Our outcomes were the changes in the following parameters between baseline and the end of the research(at least 12 months after operation): (i) Maximum keratometry value (Kmax, D): the steepest keratometry value (ii) Corrected distant visual acuity visual acuity (CDVA, logMAR): the visual acuity with correction (iii) Uncorrected distant visual acuity (UDVA, logMAR): the visual acuity without correction (iv) Thinnest corneal thickness(CT, μm): the thickness of the thinnest point. Meeting abstracts with insufficient data, duplicate publications, letters and reviews were excluded.

### Data extraction

The extraction of data from each study was performed by two authors independently. Any disagreement was discussed and resolved by the two authors. The extracted information included the name of the first author, the year of publication, the trial location, the research design, the number of eyes, the mean age of patient, interventions, the follow-up durations, and outcome measures (Kmax, CDVA, UDVA, and CT).

### Quality assessment

The methodological qualities of the included RCTs were assessed according to Cochrane Collaboration’s tool described in Handbook version 5.1.0 [20]. Two authors used this tool for assessment of study quality independently. Any disagreement was discussed by the two people and resolved. The items related to quality assessment included random sequence generation, allocation concealment, blinding of participants and personnel, blinding of outcome assessment, incomplete outcome data, selective reporting, and other biases [20].

### Statistical analysis

Considering all the included clinical characteristics were similar between groups, it was believed that there was not any obvious clinical heterogeneity. Therefore, it was reasonable to combine these studies altogether. Analyses were carried out using Review Manager Version 5.1 (The Cochrane Collaboration, Oxford, England) using 2-tailed *P* values and a 95% confidence interval (CI). For continuous outcomes, the weighted mean difference (WMD) and 95% confidence interval (CI) were calculated for absolute changes of the interested outcomes. The outcomes were measured as mean ± standard deviation (SD). Heterogeneity across studies was estimated by using X2 and I2 test (I2 > 50% indicating significant heterogeneity) [21, 22]. The overall effect was determined to be statistically significant with *P* < 0.05. Additionally, if significant heterogeneity existed among trials, a random model was used, and sensitivity analysis was conducted. Alternatively, results were combined using a fixed effect model [23].

## Results

### Literature search

**Fig. 1:**
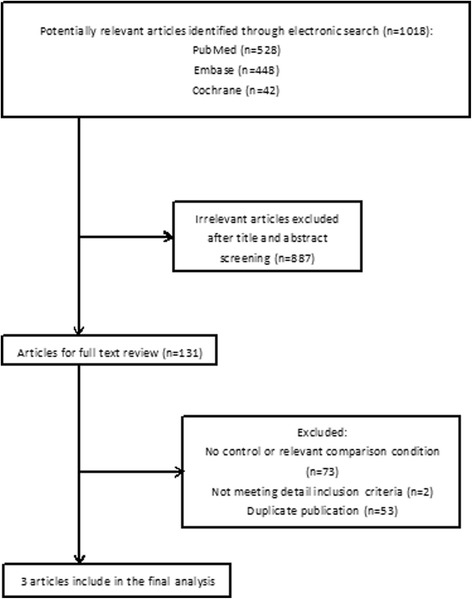
Flow diagram of study selection

As is shown in Fig. 1, there were 1018 potentially relevant articles yielded by electronic searches. Of these studies, 887 articles were excluded after screening titles and abstracts. In the remaining 131 articles, 53 reports were excluded because of duplicate publications; due to the reason of no control or relevant comparison, 73 studies were excluded; not meeting particular inclusion details, 2 articles were not included. Since Stojanovic A adopted a contralateral method to evaluate the results of the two operations which was different from other studies and this might affect each other in some way, we did not include this study in this meta-analysis. Three randomized controlled trials were included in this meta-analysis [14, 15, 18].

### Characteristics of eligible studies

The main characteristics of all eligible studies were summarized in Table 1. The studies were published in 2015 or 2016. A total of 244 eyes were evaluated, with 111 eyes in the standard CXL group and 133 eyes in the transepithelial CXL group. The included three studies were all randomized comparative design. The durations of follow-ups were 12 months, 24 months and 12 months respectively. The studies were conducted in Netherlands, Russia and Italy respectively.

**Table 1:** Characteristics of the studies included in this review

Author	Year	Country	Design	Follow-up (months)	Intervention	No. of eyes	Age(years)	∆Kmax (D)	∆CDVA (logMAR)	∆Corneal thickness (μm)
Soeters, N	2015	Netherlands	Randomized Controlled Trial	12	Standard CXL	24	24(18–44)	-1.5 ± 2.0	-0.07 ± 0.21	−4 ± 8
					Transepethelial CXL	33	24(18–48)	0.3 ± 1.8	−0.14 ± 0.21	0 ± 12
Bikbova, G	2016	Russia	Randomized Controlled Trial	24	Standard CXL	73	30(18–42)	−1.89 ± 3.023	−0.02 ± 0.2793	−13 ± 37.2252
					Transepethelial CXL	76	28(18–44)	−0.74 ± 3.0494	−0.07 ± 0.4525	−6.72 ± 38.61118
Lombardo, M	2017	Italy	Randomized Controlled Trial	12	Standard CXL	12	29.4 ± 5.6	−0.82 ± 1.20	−0.03 ± 0.06	−1 ± 494.60
					Transepethelial CXL	22	31.0 ± 6.6	−0.52 ± 1.30	−0.10 ± 0.12	9 ± 489.81

### Quality assessment

**Fig. 2:**
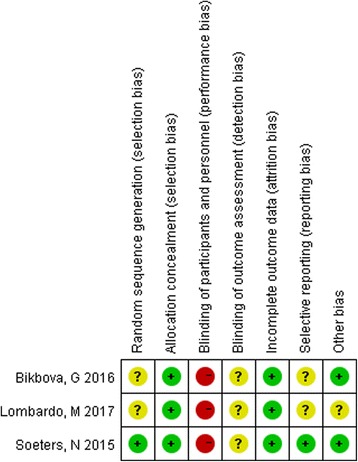
Risk of bias assessment. Risk of bias summary: review authors’ judgments about each risk of bias item for each included study

The quality assessment is shown in Fig. 2. For selection bias, 1 study used a simple unrestricted randomization procedure [15], while the other 2 studies did not state the randomization method explicitly [14, 18]. As there were obvious differences existing between the two surgical procedures, the included studies were all judged to be at high risk bias in performance bias. Blinding of outcome assessments were not clearly stated in all the studies. As to the attribution bias, only 1 study reported that 2 in each group did not complete the follow-up [15], while the other 2 studies completed without missing participants [14, 18]. For reporting bias, only 1 study were considered to be at low risk, while other two studies were not clear since no protocols were given in the articles.

### Efficacy analysis

**Fig. 3:**
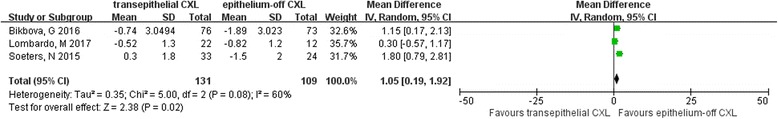
Change in the maximum keratometry value between transepithelial CXL and standard CXL groups

Maximum keratometry (Kmax). Data for Kmax were collected from all three included trials. They all favored the standard CXL group which can have a more reduction in kmax, and the meta-analysis of pooled data showed statistically significant differences between the two groups (mean difference = 1.05D, 95% CI 0.19 to 1.92, *P* = 0.02) (Fig. 3).

**Fig. 4:**
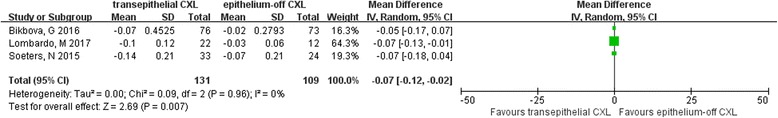
Change in the corrected distant visual acuity between transepithelial CXL and standard CXL groups

Corrected distant visual acuity (CDVA). There were 3 outcomes illustrated in the 3 trials as a logarithm of the minimal angle of resolution (logMAR). Examination of the forest plot showed that the differences between the two groups were significantly different (mean difference = −0.07, 95% CI -0.12 to −0.02, *P* = 0.007) and the transepithelial CXL group gained more improvement in CDVA (Fig. 4).

**Fig. 5:**
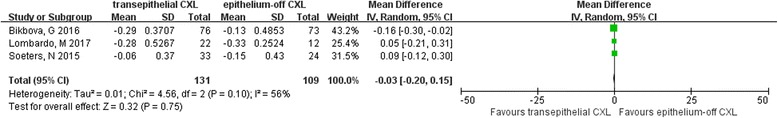
Change in the uncorrected distant visual acuity between transepithelial CXL and standard CXL groups

Uncorrected distant visual acuity (UDVA). Three studies reported data for the UDVA as a logarithm of the minimal angle of resolution (logMAR). However, examination of the forest plots revealed that the differences were not statistically significant between the two groups (mean difference = −0.03, 95% CI -0.20 to 0.15, *P* = 0.75) (Fig. 5).

**Fig. 6:**
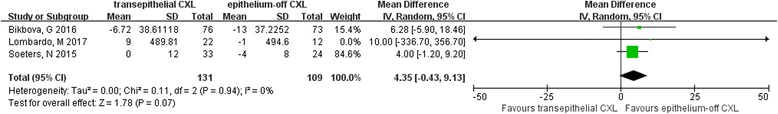
Change in the corneal thickness between transepithelial CXL and standard CXL groups

Corneal thickness on thinnest point (CT). For comparison of corneal thickness on thinnest point, data were collected in all the three trials. The pooled data showed that corneal thickness change were similar in the standard CXL group and transepithelial CXL group (mean difference = 4.35, 95% CI -0.43 to 9.13, *P* = 0.07) (Fig. 6).

## Discussion

Since Wollensak introduced the corneal collagen cross-linking (CXL) in the clinical treatment of keratoconus in 2003 [5], it has been demonstrated that CXL has shown the potential for slowing or eliminating the progression of keratoconus by a lot of researchers. However the removal of corneal epithelium still remains controversial. Thus, the transepithelial CXL is developed to avoid the adverse effects caused by removing corneal epithelium. And in recent years, several clinical trials were designed to compare the therapeutic effects between the two methods [14–19]. According to what we know, this is the first meta analysis to evaluate clinical effects and safety after standard CXL and transepithelial CXL.

Corrected distant visual acuity and uncorrected visual acuity were prudently analyzed. Based on this meta analysis, the impact of standard and transepithelial CXL on visual acuity were both remarkable. And the transepithelial CXL group gained more improvement in CDVA according to the three randomized control trials included in this analysis. However, the improvement in UDVA were similar in both groups.

In our analysis, the maximum corneal keratometry, which can indicate the progression or improvement of keratoconus, was analysized carefully. As was illustrated in the forest plot, although the two surgical procedure both decreased the maximum of corneal keratometry, the standard CXL was more effective with statistically significant difference. There are several theories behind this phenomenon. First, it is a challenge for the large hydrophilic molecule of riboflavin to penetrate the lipophilic epithelium for diffusion into the corneal stroma. On the other hand, the epithelium and the riboflavin remained in the epithelial layer can absorb the UVA, thus weakening the actual UVA power in the corneal stroma. Besides, the epithelium also acts as a barrier to oxygen diffusion to the stroma, limiting the crosslinking which happens through oxygen-dependent pathways [24–26]. Considering all these factors, the actual transepithelial crosslinking effect may be less deep and less complete at all levels compared to what occurs with equivalent dosing with the epithelium removed.

Since the reduction in the corneal thickness is a safety concern for keratoconus patients, we also pay attention to the corneal thickness on the thinnest point to assess the safety of CXL procedure. Both surgical methods lead to a similar reduction in the corneal thickness, which still remains further clinical trials to identify. The underlying mechanism is still unclear. Epithelial remodeling, compression of collagen fibrils, change in corneal hydration, and keratocyte apoptosis may play a crucial role in the process [27].

Several limitations should be taken into account when considering the results of this meta analysis. First, since there were only three randomized control trials included, the power of assessment was weakened accordingly, especially when it came to the events with low incidence rate. Only four outcomes (Kmax, CDVA, UDVA, CT) were summarized in this study. Because adequate data was unavailable, other important outcomes could not be reviewed in our meta analysis, such as spherical equivalent, intraocular pressure and endothelium cells count. Finally, this meta-analysis was restricted to data from published studies, so information bias could not be fully ruled out if studies with small sample-size or unpublished data exist. Therefore more pragmatic randomized controlled trials are needed to update this analysis.

## Conclusions

In conclusion, the effect of standard corneal collagen crosslinking on controlling the corneal keratometry is more remarkable than transepithelial corneal collagen crosslinking. With regard to visual acuity, the transepithelial CXL group gains more improvement in the corrected distant visual acuity in spite that the two surgical methods are considered to have the similar effect on the uncorrected distant visual acuity. They both are demonstrated to be safe as the corneal thickness indicated. Further RCTs are needed to confirm these findings.

## Abbreviations

CDVA: Corrected distant visual acuity
CT: Corneal thickness
CXL: Corneal collagen crosslinking
Kmax: Maximum keratometry
UDVA: Uncorrected distant visual acuity
WMDs: Weighted mean differences
